# 1373. Racial Disparities in Clinical Characteristics and Outcomes for Methicillin Susceptible and Methicillin-Resistant *Staphylococcus aureus* Bacteremia

**DOI:** 10.1093/ofid/ofab466.1565

**Published:** 2021-12-04

**Authors:** Michael C Mohnasky, Larry Park, Emily Eichenberger, Michael M Dagher, Vance G Fowler, Felicia Ruffin, Brittney N Broadnax

**Affiliations:** 1 Duke University Medical Center, Chapel Hill, North Carolina; 2 Duke University Department of Medicine, Durham, North Carolina; 3 Duke University, Durham, North Carolina

## Abstract

**Background:**

Bacterial bloodstream infections (BSI) are one of the most described syndromes in infectious diseases, but the presence of racial disparities in BSI is unclear. The purpose of this project was to determine if racial disparities exist in patients with *S. aureus* bacteremia (SAB).

**Methods:**

Data was used from a prospective cohort of patients with SAB at Duke University Medical Center from 1995-2015. Patients were categorized as African American (AA) or White. Characteristics of interest included discharge disposition, metastatic infection, persistence of SAB, and in-hospital mortality stratified by methicillin-susceptible *S. aureus* (MSSA) and methicillin-resistant *S. aureus* (MRSA) infections. Statistical comparisons were performed for binary variables with Fisher’s Exact test and continuous variables with Kruskal-Wallis test.

**Results:**

Among the 2396 patients with SAB, 1496 (62.4%) were White and 900 (37.6%) were AA. 1241 patients (51.8%) had MSSA bacteremia overall. Whites comprised 63.6% of MSSA and 61.2% of MRSA infections. AA were younger (MSSA [median, IQR]: 53.0, 44.0-64.0 vs. 62.0, 50.0-71.0, p< 0.0001; MRSA: 58.0, 46.5-69.5 vs. 64.0, 52.0-74.0, p< 0.0001) and more likely to be female (MSSA: 46.2% vs 38.2%, p= 0.007; MRSA: 53.1% vs 41.9%, p< 0.001). AA had higher rates of diabetes, hemodialysis, HIV infection for both MSSA and MRSA, but higher rates of injection drug use for MSSA only; Whites had higher rates of neoplasm, corticosteroid use, surgery for MSSA and MRSA, but higher rates of transplant for MRSA only (Figures 1, 2). AA had higher Acute Physiology Scores (MSSA: 9.0 vs 6.0, p< 0.001; MRSA: 8.0 vs 7.0, p=0.005). AA experienced increased rates of healthcare-associated infection (MSSA: 69.9% vs. 58.3%, p=0.0002; MRSA: 68.1% vs. 50.6%, p< 0.0001). Although Whites were more likely to have in-hospital mortality for MRSA (24.6 vs. 19.2, p=0.0359), discharge disposition, metastatic infection, and persistence did not vary significantly by race.

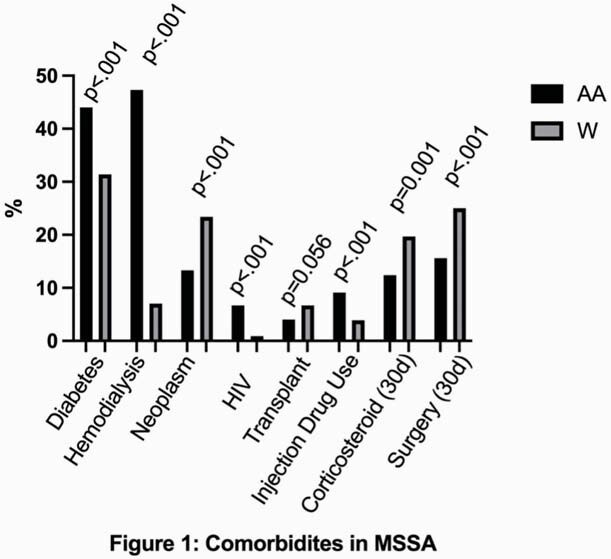

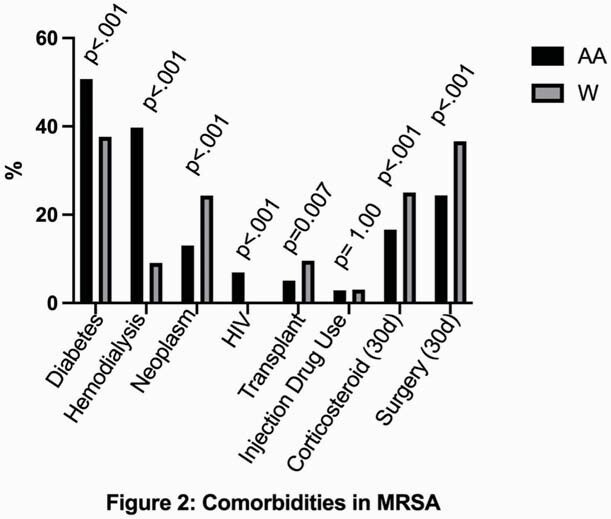

**Conclusion:**

Racial disparities exist in SAB, more so for patient characteristics than for outcomes. AA patients were younger, had a different set of comorbidities, and had more acute presentations. Although Whites had higher rates of in-hospital mortality, all other outcomes were similar.

**Disclosures:**

**Vance G. Fowler, Jr., MD, MHS**, **Achaogen** (Consultant)**Advanced Liquid Logics** (Grant/Research Support)**Affinergy** (Consultant, Grant/Research Support)**Affinium** (Consultant)**Akagera** (Consultant)**Allergan** (Grant/Research Support)**Amphliphi Biosciences** (Consultant)**Aridis** (Consultant)**Armata** (Consultant)**Basilea** (Consultant, Grant/Research Support)**Bayer** (Consultant)**C3J** (Consultant)**Cerexa** (Consultant, Other Financial or Material Support, Educational fees)**Contrafect** (Consultant, Grant/Research Support)**Debiopharm** (Consultant, Other Financial or Material Support, Educational fees)**Destiny** (Consultant)**Durata** (Consultant, Other Financial or Material Support, educational fees)**Genentech** (Consultant, Grant/Research Support)**Green Cross** (Other Financial or Material Support, Educational fees)**Integrated Biotherapeutics** (Consultant)**Janssen** (Consultant, Grant/Research Support)**Karius** (Grant/Research Support)**Locus** (Grant/Research Support)**Medical Biosurfaces** (Grant/Research Support)**Medicines Co.** (Consultant)**MedImmune** (Consultant, Grant/Research Support)**Merck** (Grant/Research Support)**NIH** (Grant/Research Support)**Novadigm** (Consultant)**Novartis** (Consultant, Grant/Research Support)**Pfizer** (Grant/Research Support)**Regeneron** (Consultant, Grant/Research Support)**sepsis diagnostics** (Other Financial or Material Support, Pending patent for host gene expression signature diagnostic for sepsis.)**Tetraphase** (Consultant)**Theravance** (Consultant, Grant/Research Support, Other Financial or Material Support, Educational fees)**Trius** (Consultant)**UpToDate** (Other Financial or Material Support, Royalties)**Valanbio** (Consultant, Other Financial or Material Support, Stock options)**xBiotech** (Consultant)

